# Blue Nevus Developing at a Combined Tetanus, Diphtheria, and Pertussis (Tdap) Vaccination Site: Case Report and Summary of Vaccine-Associated Cutaneous Lesions

**DOI:** 10.7759/cureus.4997

**Published:** 2019-06-25

**Authors:** Yoo Jung Kim, Philip R Cohen

**Affiliations:** 1 Dermatology, Stanford University, Stanford, USA; 2 Dermatology, San Diego Family Dermatology, National City, USA

**Keywords:** blue, cutaneous, deltoid, diphtheria, nevus, papule, pertussis, tetanus, vaccination, vaccine

## Abstract

Skin reaction may develop at the site of vaccine administration. A 54-year-old woman who developed a cellular blue nevus at the site of the combined tetanus, diphtheria, and acellular pertussis (Tdap) vaccine injection four years prior to presentation is described. In addition to blue nevus, other reactions at combined tetanus, diphtheria, and pertussis vaccine injection sites include abscess, deep reactive nodular infiltrates of mixed inflammation, and necrotizing granuloma. In conclusion, blue nevus can be added to the list of cutaneous events that can occur at Tdap vaccination sites.

## Introduction

Adverse cutaneous events can occur in vaccine injection sites [1]. These include inflammatory reactions and neoplasms. We describe a woman who developed a cellular blue nevus at the site of previous tetanus, diphtheria, and acellular pertussis (Tdap) injection. Cutaneous reactions to combined tetanus, diphtheria, and acellular pertussis vaccines and adverse skin reactions and neoplasms appearing in previous sites of vaccinations are also reviewed.

## Case presentation

A 54-year-old woman presented with a new lesion on her left arm. Four years prior to presentation, an acquaintance of the patient developed pertussis, which prompted the patient to seek immunization. The patient had not been vaccinated against pertussis in childhood. Based on the Center for Disease Control age recommendations, Tdap was administered on her left deltoid; the patient subsequently developed a new lesion over the previous site of the vaccine administration.

**Figure 1:**
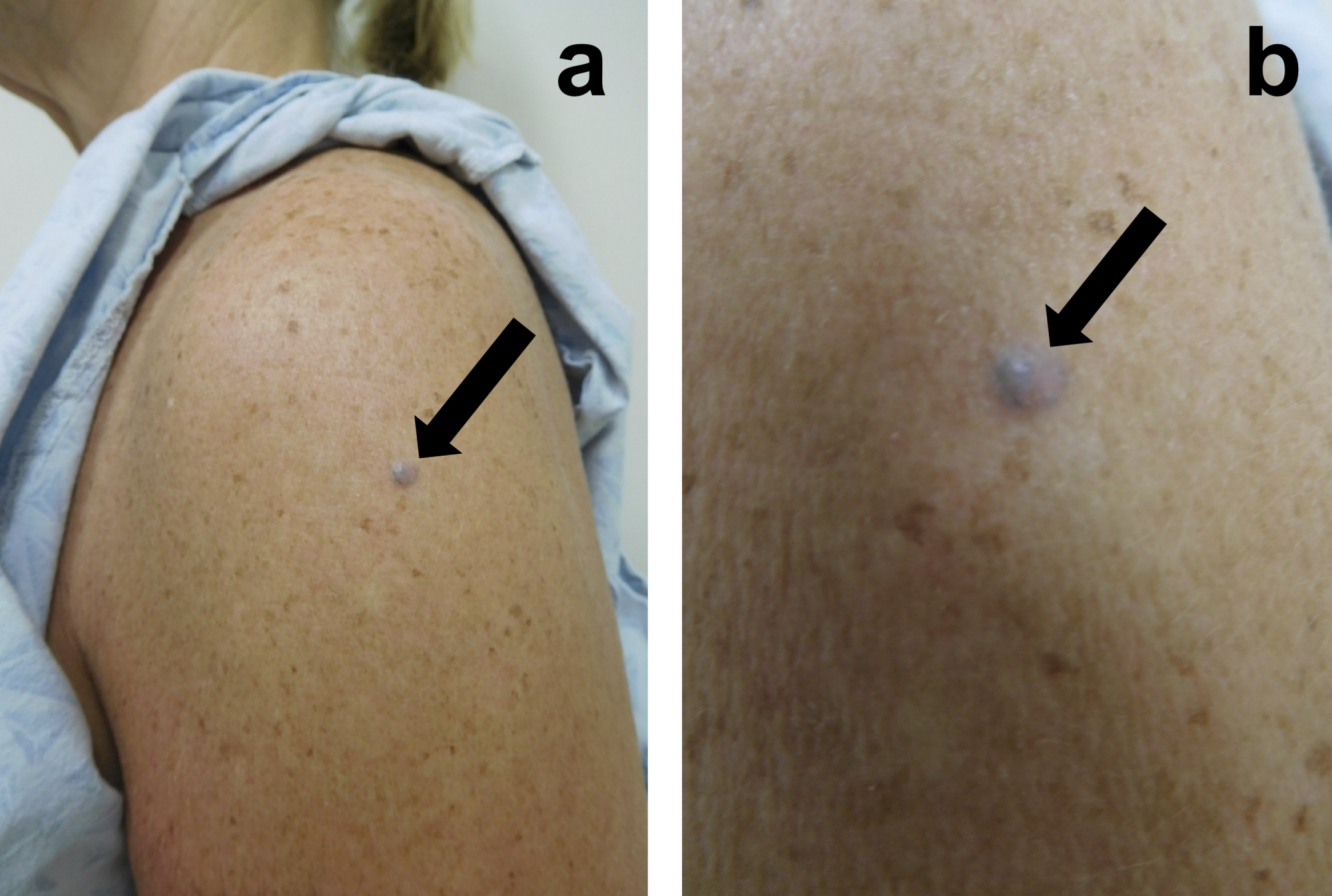
Cellular blue nevus appearing at the site of previous tetanus, diphtheria, and acellular pertussis (Tdap) vaccination Distant (a) and closer (b) views of the deltoid area of the left arm of a 54-year-old woman showing a cellular blue nevus (black arrow) that developed at the site of a previous tetanus, diphtheria, and acellular pertussis (Tdap) vaccination that she received four years earlier.

Cutaneous examination revealed a 5 x 5 mm papule on her prior Tdap vaccination site (Figure 1). A punch biopsy was performed. Microscopic examination showed ovoid melanocytes in the dermis. Collagen was trapped between the melanocytes and the surrounding fibrous stroma.

Correlation of the clinical history, lesion morphology, and pathology established the diagnosis of a cellular blue nevus developing at the site of the Tdap vaccination. The residual lesion was excised. There has been no recurrence.

## Discussion

The first vaccination against pertussis, also commonly called “whooping cough,” was formulated using killed whole-cell *Bordetella pertussis* bacilli and licensed in 1914 [2]. Currently, there is no isolated vaccination for pertussis. Combination vaccines containing diphtheria toxoids, tetanus toxoids, and chemically-inactivated whole-cell pertussis were introduced as DTP in the 1940s; the acronym DTP was based on the first letter of each respective component. Subsequently, based on concerns regarding the adverse reactions to inactivated whole-cell pertussis, researchers developed an acellular vaccination for pertussis immunity; this vaccination came into wider use in the 1990s in the United States as the combined diphtheria, tetanus, and acellular pertussis vaccination (DTaP) [2-3].

The DTaP vaccination did not maintain adequate long-term humoral immunity against pertussis [4]. Therefore, a booster formulation of tetanus toxoid and smaller concentrations of the diphtheria toxoid and acellular pertussis received the approval from the Federal Drug Administration in 2005 as Tdap. Both DTaP and Tdap are currently in use. According to the United States Center for Disease Control, in children under seven years of age, DTaP is recommended, whereas for older children and for adults who have never been vaccinated, the Tdap is recommended [5].

Numerous cutaneous reactions have been observed at the sites of both live and attenuated vaccinations. These reactions include not only inflammatory reactions such as lichenoid and granulomatous dermatosis, but also neoplasms such as basal cell carcinoma, dermatofibrosarcoma protuberans, and squamous cell carcinoma (Table 1, Table 2) [1]. Skin-related reactions specific to diphtheria, tetanus, and pertussis vaccination include deep reactive nodular infiltrates of mixed inflammation, *Mycobacterium tuberculosis* abscess, and necrotizing granuloma (Table 3) [6-7]. The development of benign or malignant neoplasms at prior sites of vaccination may be coincidental.

**Table 1:** Benign cutaneous lesions associated with vaccination sites BCG = Bacillus Calmette-Guerin vaccine; DRNIMI = Deep reactive nodular infiltrates of mixed inflammation; DTaP-IPV-Hib = Diphtheria, tetanus, pertussis, polio, Haemophilus influenzae Type B vaccine; DPT = Diphtheria, whole-cell pertussis, and tetanus vaccine; DTP = Diphtheria, tetanus, and whole-cell pertussis vaccine; ESM = Early summer meningitis vaccine; Flu = Influenza vaccine; HepB = Hepatitis B vaccine; PLAPR = Papulonodular lichenoid and pseudolymphomatous reaction; Pne = Pneumococcal vaccine; Sp = Smallpox vaccine; Td = Tetanus and diphtheria vaccine; Tdap-IPV = Tetanus, diphtheria, pertussis, and polio vaccine; Tet = Tetanus vaccine; VZV = Varicella-zoster virus vaccine ^a^Robust take is a skin reaction at the site of administration greater than 7.5 cm in size with symptoms of joint pain, swelling, and warmth.

Cutaneous lesions	Vaccination
Abscess or cellulitis	BCG, Pne
Allergic contact dermatitis	Sp
Angiolymphoid hyperplasia with eosinophils	Tet
Blistering	BCG
Churg-Strauss vasculitis	HepB
DRNIMI	DTP
Dermatitis, chronic	Sp
Dermatofibroma	Sp
Epithelial cyst	BCG
Erythema	BCG, HepB, Pne, VZV
Fixed drug eruption	BCG
Foreign body granuloma (non-necrotizing)	BCG
Granuloma annulare	BCG, HepB, Td, Tet
Granuloma (delayed)	BCG
Herpes simplex virus infection	Sp
Indurated erythematous plaque (pseudoplymphoma)	Tet
Inflammatory reaction, localized	Sp
Itching granuloma	Pne, DTaP-IPV-Hib
Isotopic response to patch testing	BCG
Keloid	BCG, HepB, Sp
Lichenoid dermatitis	Pne
Lupus erythematosus (discoid)	Sp
Lupus vulgaris (cutaneous tuberculosis)	BCG
Lymphadenopathy (suppurative)	BCG
Mastocytoma	HepB
Mycobacterium chelonae abscess	Tdap-IPV
Mycobacterium tuberculosis abscess	DPT
Myxedematous infiltration, diffuse	Sp
Necrobiotic granuloma	HepB
Necrotizing granulomatous reaction	BCG, DTP
Nevus sebaceous	Sp
Nodules	HepB
Papular tuberculids	BCG
PLAPR	HepB
Pigmentation	Sp
Pilomatricoma	BCG
Post scab lesions	Sp
Progressive vaccinia	Sp
Psoriasis	BCG, Flu
Pyogenic infections	Sp
Robust take^a^	Sp
Sarcoidosis (juvenile)	BCG
Subcutaneous nodule (sterile abscess)	DTaP-IPV-Hib
Subcutaneous nodule (pseudolymphoma)	ESM, HepB, VZV
Sweet’s syndrome	BCG, Flu, Pne, Sp
Tufted angioma	BCG
Ulceration	BCG
Ulceration during Kawasaki disease	BCG
Vasculitis (ulcerating)	BCG
Zosteriform eruption	VZV

**Table 2:** Neoplasms associated with vaccination sites BCG = Bacillus Calmette-Guerin vaccine; Lsh = Leishmaniasis vaccine; Pne = Pneumococcal vaccine; Sp = Smallpox vaccine; TPY = Tetanus, plague, yellow fever vaccine

Cutaneous lesions	Vaccination
Basal cell carcinoma	BCG, Sp
Dermatofibrosarcoma protuberans	Lsh, Sp, TPY, Travel immunization
Fibrosarcoma	Sp
Keratoacanthoma	Pne, Sp
Malignant fibrous histiocytoma	Sp
Melanoma	Sp
Squamous cell carcinoma	BCG, Sp

**Table 3:** Combined tetanus, diphtheria, and pertussis vaccine site reactions CR = Current report; DRNIMI = Deep reactive nodular infiltrates of mixed inflammation; DPT = Diphtheria, whole-cell pertussis, and tetanus vaccine; DTP = Diphtheria, tetanus, and whole-cell pertussis vaccine; Tdap = Tetanus, diphtheria, and acellular pertussis vaccine ^a^The cited reference refers to the combined diphtheria, whole-cell pertussis, and tetanus vaccination as “DPT,” which is an alternative acronym for DTP.

Reaction	Vaccination	Reference
Abscess (Mycobacterium tuberculosis)	DPT^a^	[6]
Blue nevus	Tdap	CR
DRNIMI	DTP	[7]
Necrotizing granuloma	DTP	[7]

Our patient developed a cellular blue nevus on the site of the injection after she received a Tdap vaccination [8]. To the best of our knowledge, she is the only person who has developed a vaccination site-related blue nevus. In addition, we are not aware of any other individuals developing a melanocytic lesion at the site of Tdap vaccination.

A postulated pathogenesis for the formation of a new cutaneous disease at a site of prior cutaneous insult has been attributed to the creation of an “immunocompromised district”-a localized site of immune destabilization in the skin leading to increased risk of developing dermatoses [9]. Through this potential mechanism, vaccination administration may allow the site to be more prone to developing a wide array of skin conditions. The appearance of our patient’s cellular blue nevus following localized skin trauma from the vaccination may be the etiology of this phenomenon.

The administration of vaccinations is extremely common. The development of cutaneous adverse reactions at the site of vaccination is a rare occurrence. Although the rate for cutaneous adverse reactions at the vaccination sites remains to be established, the estimated rate would be very low based on the published literature of these events.

## Conclusions

Adverse events may occur in local vaccination sites. Our patient developed a cellular blue nevus at the site of the Tdap vaccine administration. The development of a melanocytic nevus at the site of Tdap vaccination has not been previously described as a post-vaccination sequela. We suggest that this vaccination site-related tumor may have resulted from the creation of a local immunocompromised district in the skin following Tdap vaccination.
